# Spatiotemporal analysis and the characteristics of the case transmission network of 2019 novel coronavirus disease (COVID-19) in Zhejiang Province, China

**DOI:** 10.1371/journal.pone.0257587

**Published:** 2021-09-17

**Authors:** Haocheng Wu, Chen Wu, Qinbao Lu, Zheyuan Ding, Ming Xue, Junfen Lin

**Affiliations:** 1 Zhejiang Province Center for Disease Control and Prevention, Hangzhou, Zhejiang Province, China; 2 Key Laboratory for Vaccine, Prevention and Control of Infectious Disease of Zhejiang Province, Hangzhou, Zhejiang Province, China; 3 Hangzhou Centre for Disease Control and Prevention, Hangzhou, Zhejiang, Province, China; The Chinese University of Hong Kong, HONG KONG

## Abstract

**Background:**

Zhejiang Province is one of the five provinces in China that had the highest incidence of novel coronavirus disease (COVID-19). Zhejiang, ranked fourth highest in COVID-19 incidence, is located in the Yangtze River Delta region of southeast China. This study was undertaken to identify the space-time characteristics of COVID-19 in Zhejiang.

**Methods:**

Data on COVID-19 cases in Zhejiang Province from January to July 2020 were obtained from this network system. Individual information on cases and deaths was imported, and surveillance information, including demographic characteristics and geographic and temporal distributions, was computed by the system. The Knox test was used to identify possible space-time interactions to test whether cases that are close in distance were also close in time. Network analysis was performed to determine the relationship among the cases in a transmission community and to try to identify the key nodes.

**Results:**

In total, 1475 COVID-19 cases and 1 fatal case were reported from January to July 2020 in Zhejiang Province, China. Most of the cases occurred before February 15^th^, which accounted for 90.10%. The imported cases increased and became the main risk in Zhejiang Province after February 2020. The risk areas showed strong heterogeneity according to the Knox test. The areas at short distances within 1 kilometer and at brief periods within 5 days presented relatively high risk. The numbers of subcommunities for the four clusters were 12, 9, 6 and 4. There was obvious heterogeneity in the modularity of subcommunities. The maximum values of the node centrality for the four clusters were 2.9474, 4.3706, 4.1080 and 2.7500.

**Conclusions:**

COVID-19 was brought under control over a short period in Zhejiang Province. Imported infections from outside of mainland China then became a new challenge. The effects of spatiotemporal interaction exhibited interval heterogeneity. The characteristics of transmission showed short range and short term risks. The importance to the cluster of each case was detected, and the key patients were identified. It is suggested that we should focus on key patients in complex conditions and in situations with limited control resources.

## Introduction

In December 2019, a cluster of cases of pneumonia caused by severe acute respiratory syndrome coronavirus 2 (SARS-CoV-2) was reported in Wuhan, Hubei Province, China [1, 2]. Subsequently, novel coronavirus disease (COVID-19) spread into many counties [3]. On January 30, 2020, the World Health Organization (WHO) declared the outbreak a public health emergency of international concern [4]. As of August 5, COVID-19 has infected more than 18.35 million individuals worldwide and caused more than 696 000 deaths [5].

Currently, the basic epidemiological characteristics of COVID-19 are clear. The original host of SARS-CoV-2 may be bats, which spread the virus to humans through some intermediate animal hosts and caused an epidemic of COVID-19 [6]. The main source of transmission of COVID-19 is infected symptomatic patients, and carriers can also be infectious before they develop symptoms or without ever developing symptoms [6–8]. Current evidence suggests that the virus is transmitted mostly via droplets or contact [6]. The virus can also spread in poorly ventilated and/or crowded indoor settings, where people tend to spend longer periods of time. This is because aerosols remain suspended in the air or travel farther than 1 metre [9]. Fecal-oral transmission can also be a possible route [10]. The population is generally susceptible to the virus. The average incubation period is 5.2 days (95% CL: 4.1~7.0) [6].

COVID-19 remains a severe public health issue in mainland China. The top five provinces with the highest incidence are Hubei, Guangdong, Henan, Zhejiang and Hunan. Zhejiang, ranked fourth highest in COVID-19 incidence, is located in the Yangtze River Delta region of southeast China. The objective of this study is to identify the space-time characteristics of COVID-19 in Zhejiang. The Knox test was applied to identify space-time clusters and suggested a relatively high-risk boundary among a series of space-time intervals, which was compared with those in the condition of random permutation [11]. It also helps to identify the serial intervals in the epidemic with cases continually occurring [12]. We used network analysis to determine the relationships among the cases in a transmission community and tried to identify the nodes that belong to the most communities (key infection). Furthermore, the mechanism of transmission can be revealed by comparing the characteristics between different transmission communities.

## Materials and methods

### Ethical review

This study was reviewed and approved by the Ethics Committee of the Zhejiang Provincial Centers for Disease Control and Prevention. All of the data of the individuals were kept confidential. Written informed consent was obtained from all of the patients before diagnosis and reporting the data to the China Information Network System of Disease Prevention and Control. All of the methods employed in the study were in accordance with the applicable guidelines and regulations.

### Profile of Zhejiang Province

Zhejiang Province is located in southeast China between longitudes 118°E-123°E and latitudes 27°N-32°N. There are two subprovincial cities (Hangzhou and Ningbo) and nine prefecture-level cities, including Wenzhou, Huzhou, Jiaxing, Shaoxing, Jinhua, Zhoushan, Quzhou, Taizhou and Lishui, which cover 90 counties.

### The data collection

Any human COVID-19 case diagnosed in a hospital must be reported through the China Information Network System of Disease Prevention and Control by the medical staff. The data of the COVID-19 cases in Zhejiang Province from January to July 2020 were obtained from this network system. Individual information on cases and deaths was imported, and surveillance information, including demographic characteristics and geographic and temporal distributions, was computed by the system. The definition of reporting cases refers to the ‘Diagnosis and treatment of COVID-19 (trial version 7)’ [13].

### Knox test

The Knox test was carried out to detect the space-time interactions [14]. In epidemiological terms, this is usually interpreted as a test of the level of spatial clustering of infectious disease at sequential overlapping time intervals. The base Knox test statistic, X, is the number of pairs of cases that are close in both space and time. The statistic is calculated as:

$$X(s,t)=\sum_{i=2}^{N}\sum_{j=1}^{i-1}a_{ij}^{s}a_{ij}^{t}$$ (1)

where *N* is the number of cases, $a_{ij}^{s}$ is equal to 1 if cases *i* and *j* are close in space, and 0 otherwise, $a_{ij}^{t}$ is equal to 1 if cases *i* and *j* are close in time and 0 otherwise, and *s* and *t* represent prespecified spatial and temporal distances [15, 16]. To identify the serial interval of an infectious disease, the incremental Knox test (IKT) was implemented. The interval Knox statistic is formulated as:

$$IK(s,t)=\sum_{i=2}^{N}\sum_{j=1}^{i-1}a_{ij}^{s}b_{ij}^{t}$$ (2)

where $b_{ij}^{t}$ is equal to 1 if cases *i* and *j* occur *t* units apart and 0 otherwise. This result indicates which time intervals are within *t* at which spatial clustering occurs [15, 16]. This statistic is useful for testing the null hypothesis of no space-time interactions versus the alternative hypothesis of a contagious process [16]. Monte Carlo hypothesis testing, which was suggested by Mantel, is the most commonly used method for this test statistic [17]. In the Monte Carlo test, the temporal distances are randomly permuted with the spatial distances unchanged among the cases to estimate the distribution of the statistic under the null hypothesis of no space-time interaction [16]. This permutation was computed *M* times to get *M* statistics. At the same time, the *P*-value is obtained through Monte Carlo hypothesis testing, by comparing the rank of the maximum likelihood from the real data set with the maximum likelihoods from the random data sets. The *P* value is computed as:

$$\frac{R}{M+1}$$ (3)

where *R* is the rank of the real data and *M* is the number of simulations. In order for *P* to be a ‘good-looking’ number, the number of simulations is set to 999 or some other number ending in 999 such as 1999, 9999 or 99999. That way it is always clear whether to reject or not reject the null hypothesis for typical cut-off values such as 0.05, 0.01 and 0.001. Here the number of simulation was restricted to 999 and the significance was set at *p*<0.05 correspondingly.

Based on our knowledge of the COVID-19 transmission pattern and previous cluster analyses of COVID-19 transmission, the IKT is used to examine space-time interactions from 0 meters to 1000 kilometers at time intervals from 1 to 20 days [18]. The spatial distance was divided into 12 intervals. The shortest interval is 10 meters, and the longest interval is 1000 kilometers. The other intervals are 50 meters, 100 meters, 500 meters, 1 kilometer, 5 kilometers, 10 kilometers, 50 kilometers, 100 kilometers, 300 kilometers and 500 kilometers.

Furthermore, an epidemiologically meaningful notion of excess risk (ER) is calculated as the ratio of the observed statistic divided by the permutation mean *μ_x_*(*s,t*).


$$ER(s,t)=\frac{IK(s,t)}{\mu_{x}(s,t)}$$ (4)

### Transmission network analysis

Networks have become a key approach to understanding systems of interacting objects, unifying the study of diverse phenomena, including biological organisms and human society [19]. The essential element when analyzing the structure, function, and dynamics of biological networks is the identification of communities of related nodes in our study [20, 21]. This approach is greatly enhanced by clustering the links between nodes rather than the nodes themselves. With this method, it is possible for nodes to belong to multiple communities, which in turn reveals the overlapping and nested structure of the network while simultaneously identifying key nodes with membership across several communities [19, 22]. Similarities between links, *e_ik_* and *e_jk_*, that share a node (*k*) are assigned using the Jaccard coefficient, which is computed as:

$$S(e_{ik,}e_{jk})=\frac{\left|n+(i)\cap n+(j)\right|}{\left|n+(i)\cup n+(j)\right|}$$ (5)

where *n*+(*i*) refers to the first-order node neighborhood of node *i*. After assigning pairwise similarities to all of the links in the network, the links are hierarchically clustered [20]. The resulting dendrogram is cut at a point that maximizes the density of the links within the clusters after normalizing against the maximum and minimum numbers of links possible in each cluster [20].

The index of node centrality is used to measure the importance of a node in a network [21]. The node centrality can be written as:

$$C_{C}(i)=\sum_{i\in j}^{N}\left(1-\frac{1}{m}\sum_{i\in j\cap k}^{m}S(j,k)\right)$$ (6)

where the main sum is over the *N* communities to which node *i* belongs, and *S*(*j,k*) refers to the similarity between communities *j* and *k*, calculated as the Jaccard coefficient for the number of shared nodes between each community pair, and this is averaged over the *m* communities paired with community *j* and in which node *i* jointly belongs.

Furthermore, the modularity of communities—the relative number of links within the community versus links outside of the community—is computed. The modularity of community *i* can be written as:

$$M_{i}=(\frac{e_{\omega }(i)}{n_{i}(n_{i}-1)/2})*{\left(\frac{e_{b}(i)}{n_{i}\bar{d}}\right)}^{-1}$$ (7)

where *e_ω_*(*i*) and *e_b_*(*i*) are the number of links within and without community *i*, respectively, *n_i_* is the number of nodes in community *i*, and $\bar{d}$ is the average degree of nodes in the network.

## Results

### The temporal trend of the COVID-19 epidemic

**Fig 1:**
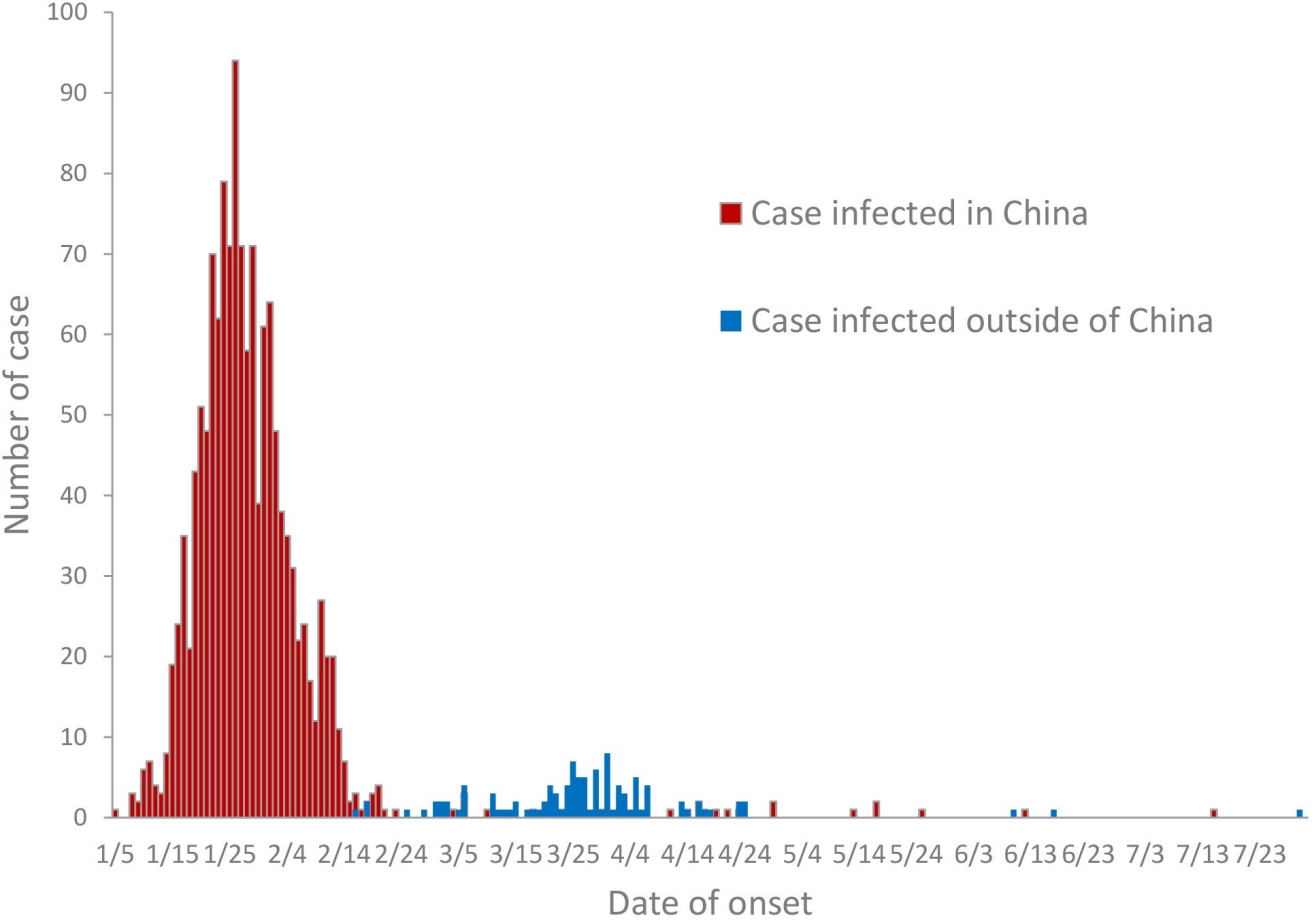
Onset of COVID-19 from January to July 2020 in Zhejiang Province, China.

From January to July 2020, there were a total of 1475 COVID-19 cases and only 1 fatal case reported in Zhejiang Province, China. The fatality rate was 0.068%. The first case was diagnosed on January 5^th^. The number of cases increased markedly starting on January 15^th^. The highest daily number of cases was 94 on January 26^th^. The incidence subsequently decreased from the beginning of February. There were 951, 398, 77, 40, 4, 3 and 2 cases identified in each month during this period. Most of the cases occurred before February 15^th^, which accounted for 90.10%. There were 1368 cases of infection inside mainland China. There were 107 cases infected outside of mainland China. The peak of the epidemic before March was attributed to mainland infections. After that, the imported cases increased and became the main risk in Zhejiang Province (Fig 1).

### Spatiotemporal patterns analysis

According to the characteristics of the temporal trend of this epidemic, only the mainland cases that occurred before March were analyzed for the spatiotemporal pattern.

Imported cases infected overseas and sporadic endemic cases after February were not included for analysis. The total number of cases used in the analysis was 1344.

**Fig 2:**
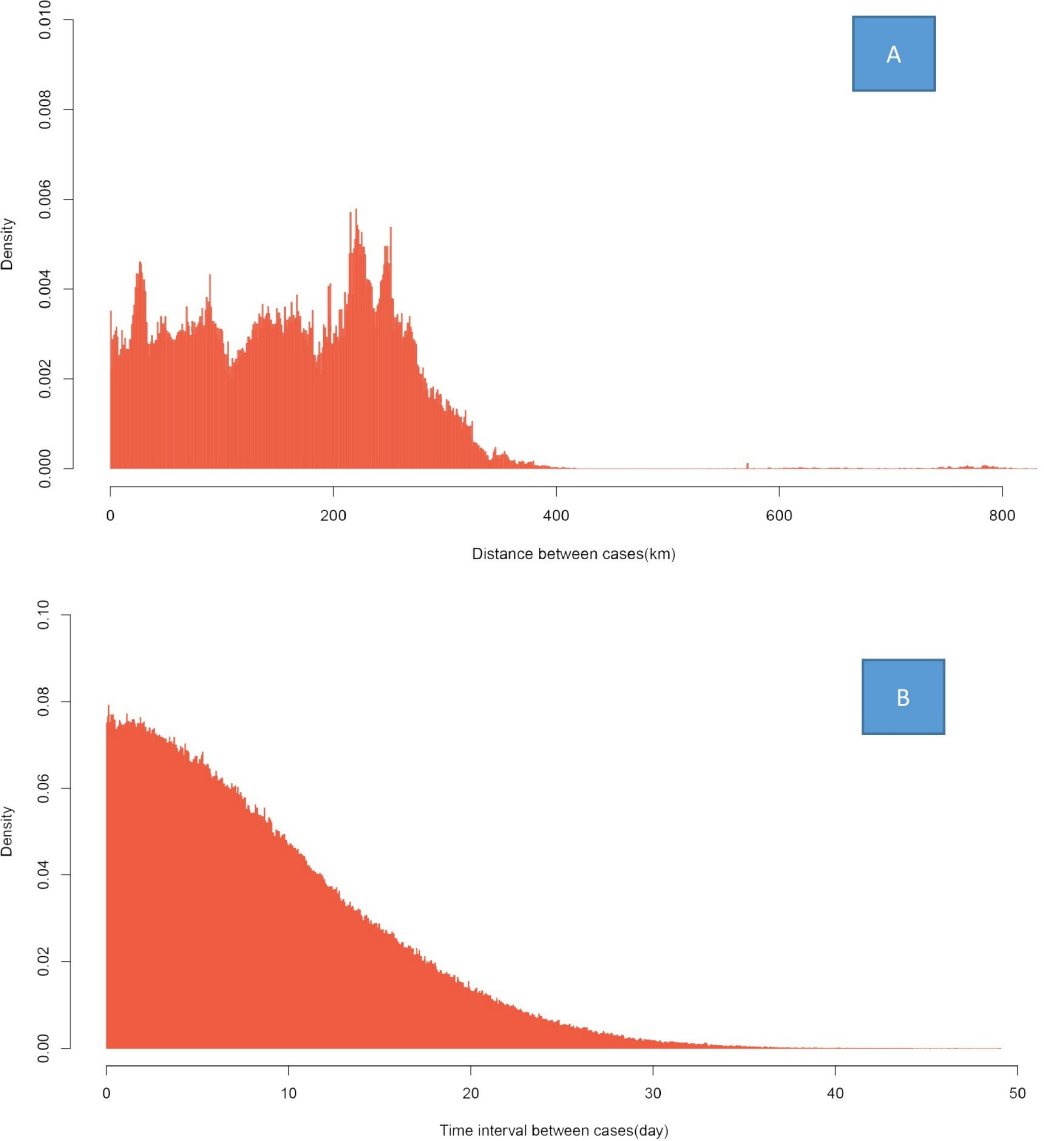
The density distribution of the interval between two COVID-19 cases during the epidemic. (A) The density of the distribution of distance between two cases. (B) The density of the distribution of the onset interval between two cases.

The median distance between the case pairs was 161.2 kilometers. The distance of most of the case pairs was within 200 kilometers. The time distances were divided into 20 intervals. The interval was 1 day. The median time interval between the case pairs was 7.23 days. The time interval of most of the case pairs was within 20 days (Fig 2).

**Fig 3:**
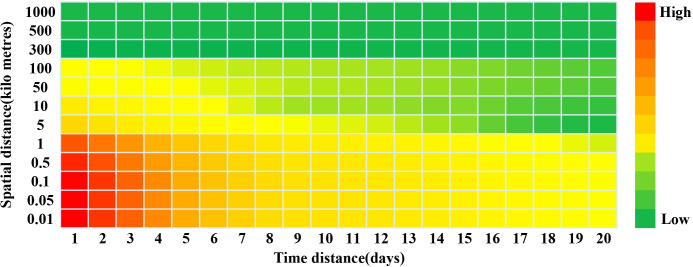
Heatmap of the excess risk due to space-time interactions for the COVID-19 epidemic in Zhejiang Province from January to July 2020.

The excess risk (ER) values are reported in Table 1. Most of the values are significantly different from zero at the alpha = 0.05 level. It should be noted that the values for the distance interval beyond 100 kilometers are not significant. The values from 1 to 5 kilometers at time intervals from 19 to 20 days are also not significant. The risk areas show a strong heterogeneity. The highest levels of elevated risk occurred at intervals of 0 and 1 day and within 10 m. The areas at short distances within 1 kilometer and at brief periods within 5 days presented relatively high risk (ER>1.5, most of the values were greater than 2). Beyond these space-time limits, the excess risk values rapidly decreased. When the distance between two cases increased, the ER values were smaller. At the same time, the ER values also gradually decreased as the onset interval between the two cases was prolonged. However, particular patterns, such as temporal periodicity of the sharp increase in risk, were not observed, the serial interval of this epidemic can’t be confirmed (Fig 3).

**Table 1:** The ER values for the epidemic of COVID-19 in Zhejiang Province using a range of distances and time intervals.

Time interval(days)	Distance(kilometers)											
	0.01	0.05	0.1	0.5	1	5	10	50	100	300	500	1000
**1**	*3*.*11*	*3*.*10*	*3*.*09*	*2*.*81*	*2*.*42*	*1*.*38*	*1*.*21*	*1*.*08*	*1*.*05*	0.99	1.00	1.00
**2**	*2*.*69*	*2*.*68*	*2*.*68*	*2*.*46*	*2*.*16*	*1*.*28*	*1*.*15*	*1*.*07*	*1*.*05*	1.00	1.00	1.00
**3**	*2*.*34*	*2*.*33*	*2*.*32*	*2*.*15*	*1*.*90*	*1*.*21*	*1*.*12*	*1*.*06*	*1*.*05*	1.00	1.00	1.00
**4**	*2*.*01*	*2*.*00*	*2*.*00*	*1*.*87*	*1*.*68*	*1*.*16*	*1*.*09*	*1*.*06*	*1*.*05*	1.00	1.00	1.00
**5**	*1*.*73*	*1*.*73*	*1*.*73*	*1*.*64*	*1*.*50*	*1*.*12*	*1*.*07*	*1*.*05*	*1*.*04*	1.00	1.00	1.00
**6**	*1*.*55*	*1*.*55*	*1*.*55*	*1*.*49*	*1*.*39*	*1*.*09*	*1*.*05*	*1*.*04*	*1*.*04*	1.00	1.00	1.00
**7**	*1*.*43*	*1*.*43*	*1*.*43*	*1*.*39*	*1*.*30*	*1*.*07*	*1*.*04*	*1*.*04*	*1*.*04*	1.00	1.00	1.00
**8**	*1*.*34*	*1*.*34*	*1*.*34*	*1*.*31*	*1*.*24*	*1*.*06*	*1*.*04*	*1*.*04*	*1*.*04*	1.00	1.00	1.00
**9**	*1*.*29*	*1*.*30*	*1*.*30*	*1*.*27*	*1*.*21*	*1*.*05*	*1*.*03*	*1*.*03*	*1*.*03*	1.00	1.00	1.00
**10**	*1*.*28*	*1*.*28*	*1*.*28*	*1*.*26*	*1*.*20*	*1*.*05*	*1*.*03*	*1*.*03*	*1*.*03*	1.00	1.00	1.00
**11**	*1*.*27*	*1*.*27*	*1*.*27*	*1*.*24*	*1*.*19*	*1*.*04*	*1*.*03*	*1*.*03*	*1*.*03*	1.00	1.00	1.00
**12**	*1*.*25*	*1*.*25*	*1*.*25*	*1*.*22*	*1*.*17*	*1*.*04*	*1*.*03*	*1*.*03*	*1*.*03*	1.00	1.00	1.00
**13**	*1*.*22*	*1*.*22*	*1*.*22*	*1*.*20*	*1*.*15*	*1*.*04*	*1*.*03*	*1*.*03*	*1*.*03*	1.00	1.00	1.00
**14**	*1*.*20*	*1*.*20*	*1*.*20*	*1*.*18*	*1*.*13*	*1*.*03*	*1*.*02*	*1*.*03*	*1*.*03*	1.00	1.00	1.00
**15**	*1*.*17*	*1*.*17*	*1*.*17*	*1*.*15*	*1*.*11*	*1*.*03*	*1*.*02*	*1*.*02*	*1*.*02*	1.00	1.00	1.00
**16**	*1*.*14*	*1*.*14*	*1*.*14*	*1*.*12*	*1*.*09*	*1*.*02*	*1*.*02*	*1*.*02*	*1*.*02*	1.00	1.00	1.00
**17**	*1*.*11*	*1*.*12*	*1*.*11*	*1*.*10*	*1*.*07*	*1*.*01*	*1*.*01*	*1*.*02*	*1*.*02*	1.00	1.00	1.00
**18**	*1*.*09*	*1*.*09*	*1*.*09*	*1*.*08*	*1*.*05*	*1*.*01*	*1*.*01*	*1*.*02*	*1*.*02*	1.00	1.00	1.00
**19**	*1*.*08*	*1*.*08*	*1*.*08*	*1*.*07*	*1*.*05*	1.00	*1*.*01*	*1*.*01*	*1*.*01*	1.00	1.00	1.00
**20**	*1*.*06*	*1*.*07*	*1*.*07*	*1*.*06*	*1*.*04*	1.00	*1*.*01*	*1*.*01*	*1*.*01*	1.00	1.00	1.00

The number in italic font indicates that the ER value is significantly different from zero at the alpha = 0.05 level.

### Transmission network analysis

There were 198 clusters from January to July 2020 in Zhejiang Province. The number of infections in most of the clusters (51.51%) was only 2. The number of cases in seven clusters was more than 15. Furthermore, the transmission relationship among the cases was explicit only in four clusters; thus, transmission networks were built for these four clusters. The numbers of affected cases in these four clusters were 66, 23, 23 and 19, respectively.

**Fig 4:**
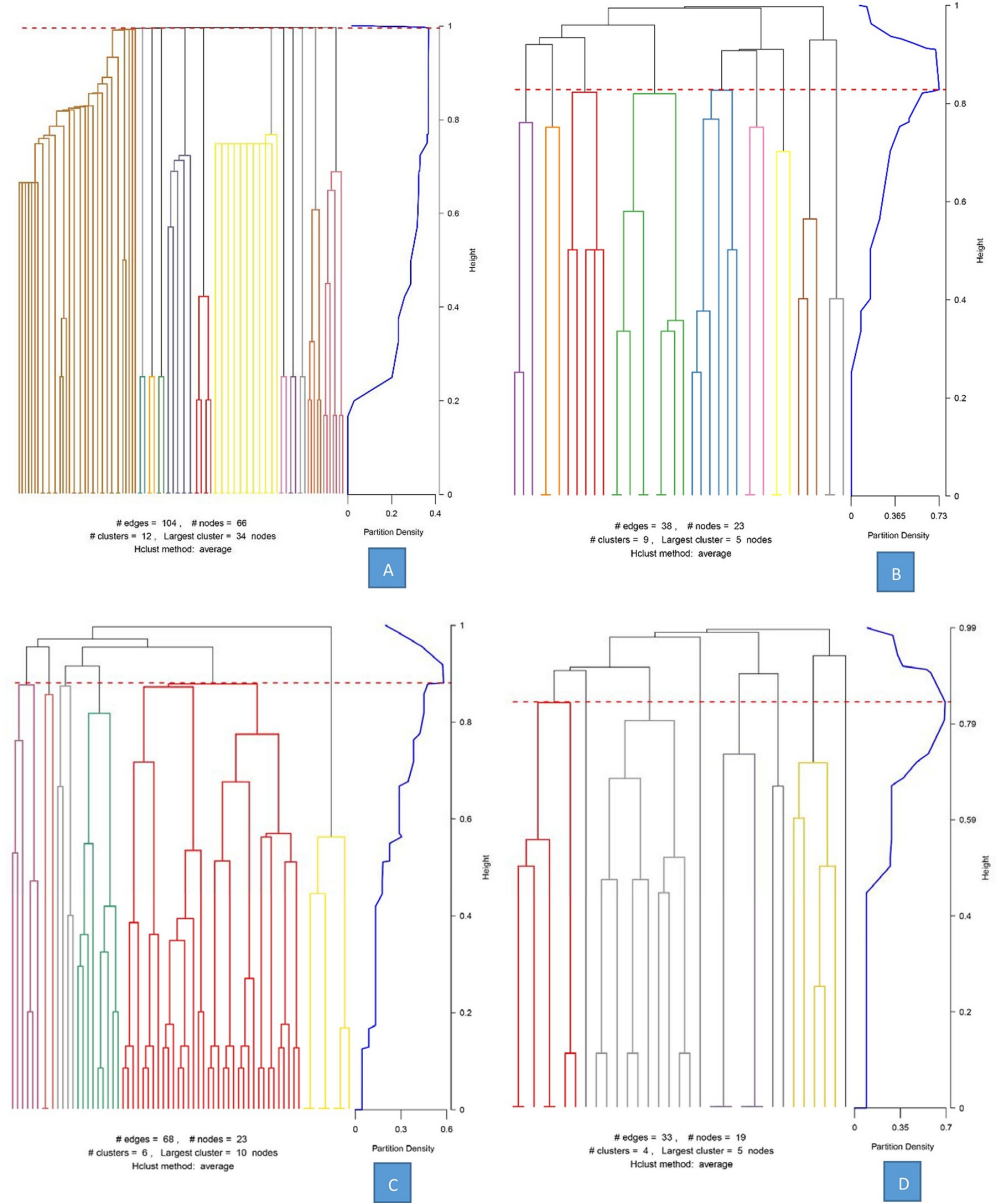
The dendrogram of link clustering for the clusters. (A) The dendrogram of cluster 1. (B) The dendrogram of cluster 2. (C) The dendrogram of cluster 3. (D) The dendrogram of cluster 4.

In the first step, dendrograms were built by the link clustering algorithm. This plot shows the link communities that result from cutting the dendrogram at a point where the partition density is maximized (Fig 4). In this step, each cluster was divided into several subcommunities. It will produce the network of the clusters. In cluster one, which included 66 infections, 104 edges and 66 nodes were identified in this network. This cluster was finally divided into 12 subcommunities, and the largest subcommunities contained 34 nodes (Fig 4A). In cluster two, which included 23 infections, there were 38 edges and 23 nodes identified in this network. This cluster was finally divided into 9 subcommunities, and the largest subcommunities contained 5 nodes (Fig 4B). In cluster three, which included 23 infections, 68 edges and 23 nodes were identified in this network. This cluster was finally divided into 6 subcommunities, and the largest subcommunities contained 10 nodes (Fig 4C). In cluster four, which included 19 infections, there were 33 edges and 19 nodes identified in this network. This cluster was finally divided into 4 subcommunities, and the largest subcommunities contained 5 nodes (Fig 4D).

**Fig 5:**
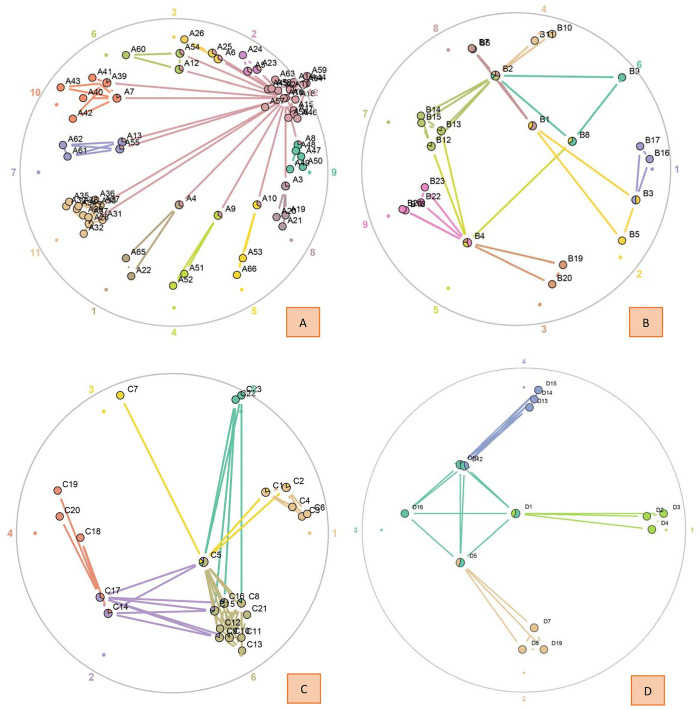
The network of transmission between cases. (A) The network of cluster 1. (B) The network of cluster 2. (C) The network of cluster 3. (D) The network of cluster 4. The fraction of the total number of edges that a node has in each community is depicted using a pie chart. The colorful numbers outside of the circle indicate the serial number of each subcommunity.

In the second step, networks were built, and the relationship between the cases was plotted (Fig 5). In cluster one, the maximum value of node centrality was identified in case A8, with a centrality of 2.9474 (Fig 5A). The node centralities of cases A4, A9, A10 and A3 were 2.9444, 2.9444, 2.9444 and 2.9459, respectively. The node centrality of these cases mentioned above was almost equal, which suggested that these cases were similarly important for transmission in cluster 1. The maximum value of the subcommunities to which these cases belong is 2. In cluster two, the maximum value of node centrality was identified in case B2, with a centrality of 4.3706, followed by B4 (3.5644), B1 (2.6667) and B8 (2.6667). The maximum number of subcommunities to which these cases belong is 4 (Fig 5B). Case B2 is the most important transmission node in cluster 2. In cluster three, the maximum value of node centrality was identified for case C5, with a centrality of 4.1080, followed by C15 (2.9500) and C16 (2.9500). Case C5 also had the maximum value of subcommunities (4), which means that C5 is the most important node in cluster 3 (Fig 5C). In cluster four, cases D1 and D5 had the maximum value of node centrality (2.7500), followed by cases D10 (2.5000) and D12 (2.5000). The maximum number of subcommunities to which these cases belong is 2 (Fig 5D).

In the third step, the modularity of the subcommunity was calculated. There was obvious heterogeneity in the modularity of the subcommunities. In cluster one, the minimum value of the modularity of the subcommunities was 0.13 for subcommunity 12. This result suggested that the number of case links related to other subcommunities is greater than the links inside this subcommunity. In cluster two, the minimum value of the modularity of subcommunities was 0.44 for subcommunity 5. In cluster three, the minimum value of the modularity of subcommunities was 0.45 for subcommunity 2. Furthermore, the minimum value of the modularity of subcommunities was 1.09 for subcommunity 3 in cluster four (Table 2).

**Table 2:** The modularity of the subcommunity of the COVID-19 clusters in Zhejiang Province.

Cluster	Subcommunity											
	No.1	No.2	No.3	No.4	No.5	No.6	No.7	No.8	No.9	No.10	No.11	No.12
**No.1**	9.45	4.73	4.73	9.45	9.45	4.73	6.30	10.51	11.03	5.67	6.02	0.13
**No.2**	4.96	1.98	1.42	1.10	0.44	0.99	1.65	1.10	1.98	-	-	-
**No.3**	14.78	0.45	0.59	1.31	0.47	2.96	-	-	-	-	-	-
**No.4**	2.32	2.32	1.09	1.52	-	-	-	-	-	-	-	-

## Discussion

According to the temporal characteristics of the seven-month epidemic, we found that COVID-19 was brought under control over a short period in Zhejiang Province. Actually, this disease was incorporated as a notifiable disease in the Infectious Disease Law and Health and Quarantine Law in China on January 20^th^ [2]. The first case in Zhejiang Province was reported on January ^21^, and then the control measures were strengthened after that date [23]. Therefore, took nearly one month from January 21^st^ to February 15th to eliminate the local epidemic. The result of Knox test suggested that the transmission pattern showed short-range and short-term risk. This trend was similar to the epidemic curve in other provinces of China [24]. There were no effective pharmacological interventions or vaccines available at that time, so reducing the rate of infection was a priority and the prevention of infection was the best approach to flatten the epidemic curve [25]. According to the experience of disease control measures implemented in Zhejiang Province and other provinces of China, the most effective approach to preventing disease transmission is expanding the physical or social distance and the use of face masks, which allowed the epidemic to be slowed down over a short period [24]. The result of Zhejiang Province give an strong evidence that timely non-pharmaceutical interventions are essential to quickly control the epidemic. The fatality rate in Zhejiang Province was lower than the average rate in China [24]. The main reason may be the high urbanization rate and GDP (gross domestic product) per capita in Zhejiang Province (ranked fourth in China with 15,755 dollars in 2019). These also reflect high-level medical conditions that led to a low fatality rate. After containing the endemic infections, imported infections from outside of mainland China became another challenge. The subsequent preparedness and response to this pandemic should continue in all countries/territories/areas worldwide [26].

The spatiotemporal analysis of the Knox test has been widely applied to dengue fever, cerebrospinal meningitis, and hand, foot, and mouth disease [11, 12, 27, 28]. This method helped us to identify the boundaries in space and time of the maximum contagious disease transmission. It also allowed us to identify high-risk areas with precise distances and time intervals. Furthermore, temporal periodicity can be detected during continuous transmission conditions [11, 12]. In our study, the effect of a spatiotemporal interaction was obvious and exhibited interval heterogeneity. The principal finding in our study is that the areas with the highest level of elevated risk were all within a distance of 1 kilometer. The elevated risk of clustering in this period (January 1^st^ to March 31^st^) of the epidemic in Zhejiang Province was relatively high within 5 days. The highest risk value occurred at intervals of 0 and 1 day and gradually decreased as the onset interval was prolonged. The characteristics of transmission in these periods showed short-range risk, and the risk of remote transmission was relatively low. This result suggested that COVID-19 infection from one case to other individuals is inclined to occur in nearby persons. According to previous studies, the main reason for this short-range high risk is that family clusters accounted for a significant number of infections during that period of the COVID-19 epidemic in China [29–31]. Most of the cases came from the same family that lived in one house or near each other. Furthermore, the timing of the outbreak, prior to the annual Chinese Lunar New Year holiday, resulted in a higher frequency of having dinner parties among family members [4]. The cases who returned from Wuhan city took part in family dinners and then transmitted the disease to other family members who usually became the secondary case.

As the intervention measures were only implemented in late January, at that time, the social distance was lengthened and social transmission was interrupted since the risk of remote range transmission was lower than that of short range transmission. At the same time, the characteristics of the heatmap of the excess risk also indicated that the epidemic curve declined rapidly over one month. Another reason may be that individuals often wear face masks to protect themselves against infection by this virus when they are outside. However, they generally do not use them at home or in their own neighborhood, which leads to more short range transmission. Unlike previous studies on dengue fever, temporal periodicity was not identified in our study. The risk values decreased as the onset interval was prolonged. The high-risk areas were within one serial interval. The reason for this should be attributed to timely interventions to block the spread of the disease at the social level and to confine most cases within family clusters. This result also suggested that the timely non-pharmaceutical interventions are essential to quickly control the epidemic.

According to the results of the transmission network analysis, we found that the importance to the cluster of each case was different. A few cases became important transmission nodes due to their high value of node centrality. This result suggested that these cases played a key role in the cluster, and some of them, such as case C5, may be a superspreader who transmits the virus to more than 10 persons. Why did these cases become key nodes in the transmission network? Careful investigation should be launched in the future to address this problem. Nevertheless, based on previous studies, the factors for a case becoming a source of infection of COVID-19 include the occupation of the case, their behavior and living habits, the range of their activity, their state of illness and so on [32]. Furthermore, an increasing number of studies have indicated that 30~60% of infections are asymptomatic and mild cases [33, 34]. These asymptomatic and mild cases can also transmit the virus to other people [33–35]. Because it is difficult to distinguish these cases from healthy persons, the chance of spreading the virus by asymptomatic and mild cases would be greater than that of severe patients. Therefore, these cases could also become key nodes in the transmission network.

In addition to the key nodes in the network, an ability to spread the virus to other subcommunities or subclusters was detected. The heterogeneity in the modularity of subcommunities suggested that a group of cases played a more important role in the network to spread the virus to other persons and lead to secondary infections. Based on the importance of transmission within the cluster for each case, we should focus on the key patients (key nodes in the network) in complex conditions or situations of limited control resources. When key patients are brought under control, the main transmission chain can be interrupted, which will help to mitigate the epidemic.

Several limitations should be noted within our study. First, in the spatiotemporal analysis, the distance between the cases was computed by their usual living address. However, the actual site where the case contracted the virus may be a working location or a public place, such as mass transit and restaurants. Therefore, the risk areas of the distance interval may be biased. Second, our sample included cases of COVID-19 reported from a passive surveillance system. Future studies should consider mild and asymptomatic cases that do not seek medical care. Third, data regarding the population characteristics, including socioeconomic status, human activities and clinical progress, were not collected. Consequently, the difference between the case as the key node and the other cases in the transmission network could not be compared. To achieve an accurate forecast and timely intervention for the possible key patient in transmission, future studies should incorporate these factors into the analysis.

In conclusion, COVID-19 was brought under control over a short period in Zhejiang Province. The fatality rate in Zhejiang Province was lower than the average rate in China. Imported infections from outside of mainland China then became a new challenge. Based on the Knox test, the effect of spatiotemporal interactions exhibited interval heterogeneity. The areas with the highest level of elevated risk were within a distance of 1 kilometer and a period of 5 days. The characteristics of transmission showed short-range and short-term risk. The pattern of non-temporal periodicity suggested that timely non-pharmaceutical interventions are essential to quickly control the epidemic. Using transmission network analysis, the importance to the cluster of each case was detected, and the key patients were identified. It is suggested that we should focus on key patients in complex conditions or in situations with limited control resources.

## Supporting information

S1 File The database of the COVID-19 cases in Zhejiang Province. (CSV)
